# Alveolar Bile and Light Chain Immunoglobulin Depositions as an Unusual Complication of Transjugular Liver Biopsy Resulting in Bilhemia in a Patient with Multiple Myeloma

**DOI:** 10.3390/jcm14061871

**Published:** 2025-03-11

**Authors:** Silvia Farkašová Iannaccone, Sylvia Dražilová, Radoslav Matěj, Miroslava Takáčová, Peter Bohuš, Peter Jarčuška, Adriána Šmirjáková, Alžbeta Ginelliová, Lucia Fröhlichová, Štefan Pataky, Miloš Kička, Zuzana Szamosi, Daniel Farkaš

**Affiliations:** 1 Department of Forensic Medicine, Faculty of Medicine, Pavol Jozef Šafárik University, Trieda SNP 1, 041 11 Košice, Slovakia; 2 2nd Department of Internal Medicine, Pavol Jozef Šafárik University, Louis Pasteur University Hospital, Trieda SNP 1, 040 11 Košice, Slovakia; 3 Department of Pathology and Molecular Medicine, 3rd Faculty of Medicine, Charles University, Thomayer University Hospital, Vídeňská 800, 140 59 Prague, Czech Republic; radoslav.matej@ftn.cz; 4 Department of Pathology, Louis Pasteur University Hospital, Rastislavova 43, 040 01 Košice, Slovakiapeterbohus9@gmail.com (P.B.); smirjakova@yahoo.com (A.Š.);; 5 Medicolegal Department of Health Care Surveillance Authority, Ipeľská 1, 043 74 Košice, Slovakia; 6 Department of Radiodiagnostics and Imaging Techniques, Pavol Jozef Šafárik University, Trieda SNP 1, 040 11 Košice, Slovakia; 7 1st Department of Surgery, Louis Pasteur University Hospital, Rastislavova 43, 040 01 Košice, Slovakia

**Keywords:** transjugular liver biopsy, bilhemia, multiple myeloma, diffuse alveolar damage, alveolar bile deposition, alveolar light chain deposition

## Abstract

**Background:** A 69-year-old man with multiple myeloma and left-sided heart failure presented to the hospital with a two-month fever. **Method:** A transjugular liver biopsy was performed due to the rapid progression of liver failure. The procedure was complicated by an intraperitoneal hemorrhage. The bleeding was managed expectantly. **Result:** Significantly elevated serum bilirubin levels occurred on the 13th day after liver biopsy. Increasing serum bilirubin levels were observed until the patient’s death due to a biliovenous fistula at the liver biopsy site. Simultaneously, his slightly elevated liver enzymes returned to normal. The patient died 23 days after liver biopsy due to acute respiratory distress syndrome. Fistulous communication between the biliary tree and the hepatic venous system with subsequent bile leakage into the venous system (bilhemia) can lead to bile deposition in the lungs. Bile deposition in the lungs may potentiate and accelerate the development of diffuse alveolar damage with hyaline membranes. **Conclusions:** Lambda and kappa light chain deposition in the pulmonary alveoli in patients with multiple myeloma can mimic typical hyaline membranes.

## 1. Introduction

Transjugular liver biopsy (TJLB) is usually performed in patients with suspected diffuse parenchymal disease and when coagulopathy or ascites prevent the safe performance of a percutaneous liver biopsy. TJLB is indicated for patients with coagulopathy, ascites, peliosis hepatis, morbid obesity, and after liver transplant. TJLB was first described by Dotter in 1964 and clinically performed for the first time by Hanafee in 1967 [1]. The overall complication rate for TJLB is low. Compared to the adult population, complications in the pediatric population are much more common [2]. Reported mortality is 0.09% in adults. Reported causes of death include intraperitoneal hemorrhage (0.06%) and ventricular arrhythmias (0.03%) [3]. The complication rate is lower for TJLB than for percutaneous or mini-laparoscopic liver biopsies [4].

## 2. Case Description

A 69-year-old man (height: 175 cm, weight: 65 kg, body mass index: 21.2) with diabetes and left-sided heart failure is described in this case report. He was treated for multiple myeloma IgG lambda IIIA for one year prior to being readmitted to the hospital. At the time of diagnosis, he had a lambda light chain level of 161.25 mg/L and a kappa light chain level of 15.30 mg/L. One year later, when he was readmitted to the hospital, the lambda light chain level was 844.26 mg/L (normal range: 5.7–26.3 mg/L), and the kappa light chain level was 36.16 mg/L (normal range: 3.3–19.4 mg/L). The anicteric patient presented to the hospital with a fever of two months and clostridial enterocolitis, which were managed successfully. Abnormal liver tests were inconclusive; therefore, a liver biopsy was performed to definitively elucidate the cause of the permanently elevated liver enzymes. The biopsy result was thought to provide the key piece of information with respect to this unexplained condition. The differential diagnosis primarily included tumor involvement. A different type of cancer would have required a completely different therapeutic approach. Due to severe thrombocytopenia and secondary coagulopathy, TJLB was performed. The patient was sufficiently informed about the procedure and its potential risks (e.g., hemorrhage). The predictable risk factors (age, compromised health condition) for an adverse event were discussed. Liver biopsy also excluded the diagnosis of possible hemophagocytic lymphohistiocytosis, a condition which was also considered in the differential diagnosis of liver injury.

Liver tissue samples were obtained using the right jugular and right hepatic vein access. After the procedure, the patient complained of pain under the right side of his rib cage. Abdominal ultrasound revealed that blood had leaked from the biopsy site into the peritoneal cavity.

Surgical revision of the abdominal cavity was required due to bleeding from the left hepatic vein after liver biopsy. The postoperative period was uneventful. The liver biopsy revealed minimal inflammatory activity, hepatic sinus congestion, and intracellular cholestasis. Immunohistochemistry showed no signs of tumor infiltration and appeared to be lambda-light-chain-negative. A repeat bone marrow evaluation revealed no evidence of disease progression. The patient died three weeks after liver biopsy. Death was attributed to acute respiratory distress syndrome (ARDS). The cause of the intracellular cholestasis was not elucidated.

**Figure 1:**
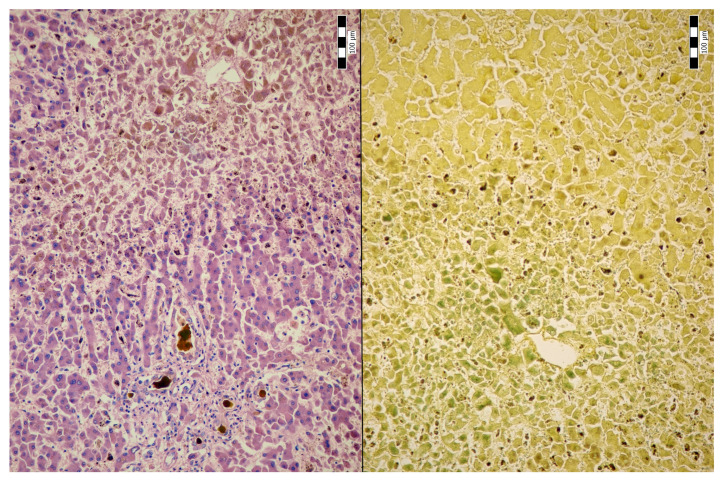
Intrahepatic cholestasis (H & E, 200x/left and Fouchet’s reagent, 200x/right).

**Figure 2:**
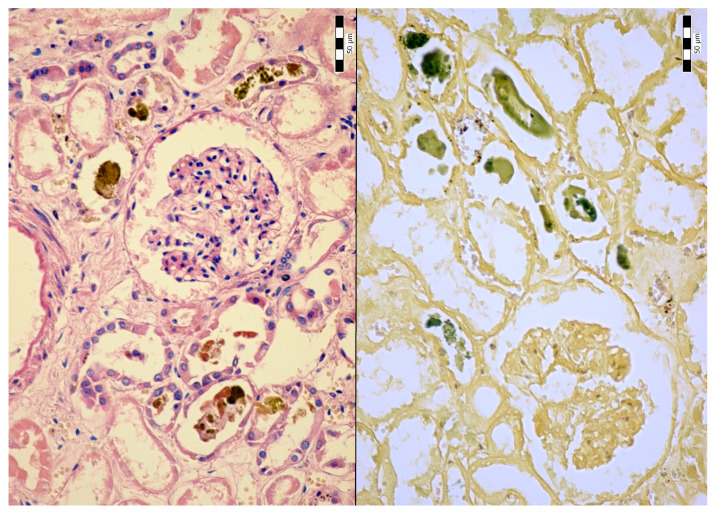
Cholemic nephrosis (H & E, 200x/left and Fouchet’s reagent. 200x/right).

**Figure 3:**
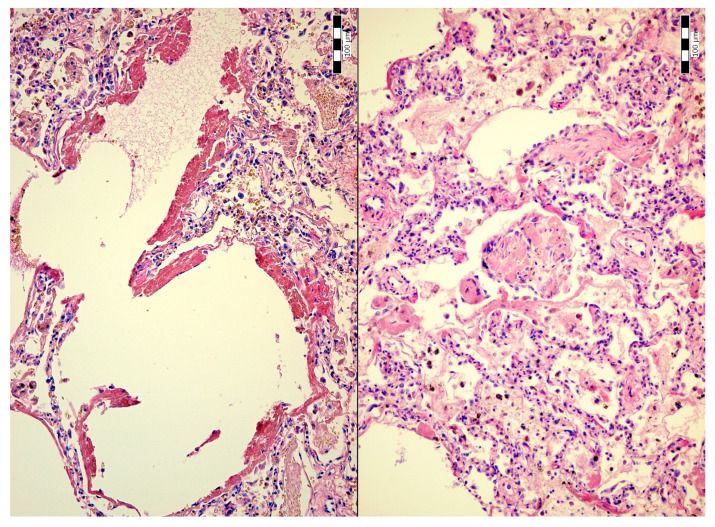
Diffuse alveolar damage: hyaline membranes (H & E, 200x/left) and proliferative phase (H & E, 200x/right).

**Figure 4:**
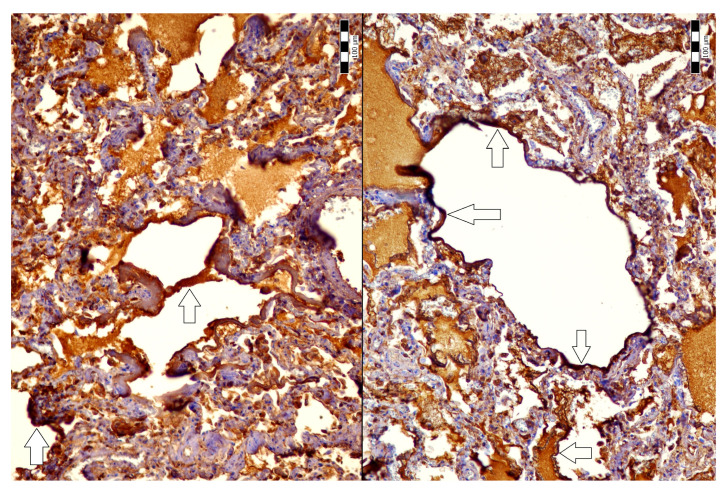
Positive immunohistochemical stain for lambda light chains in the pulmonary alveoli (200x, arrows).

**Figure 5:**
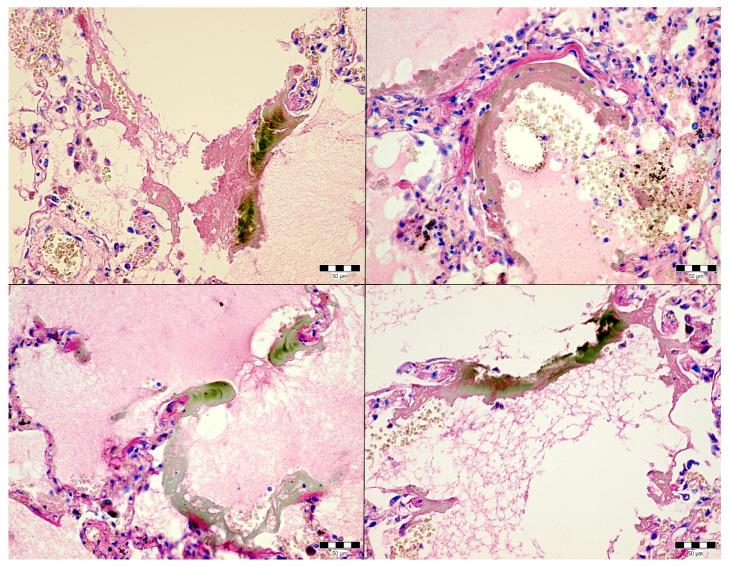
Alveolar bile deposition in the lungs (H & E, 400x).

**Figure 6:**
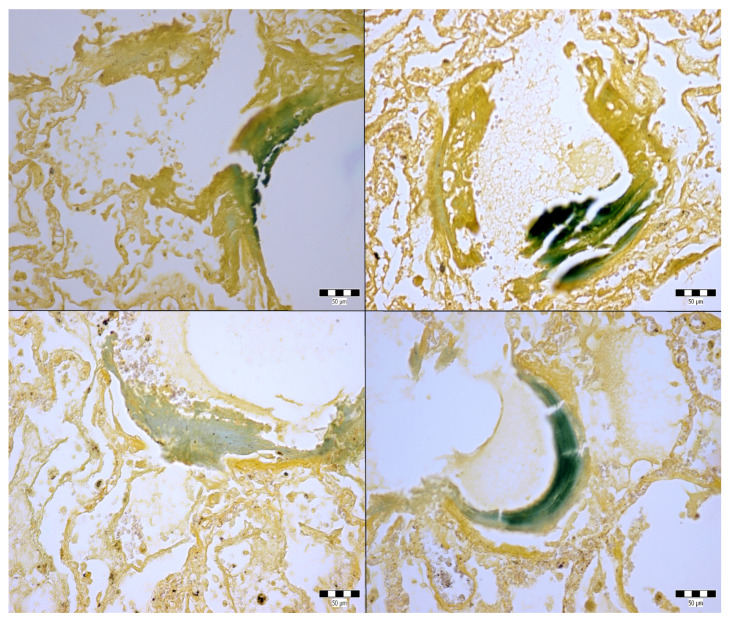
Alveolar bile deposition in the lungs (Fouchet’s reagent, 400x).

**Figure 7:**
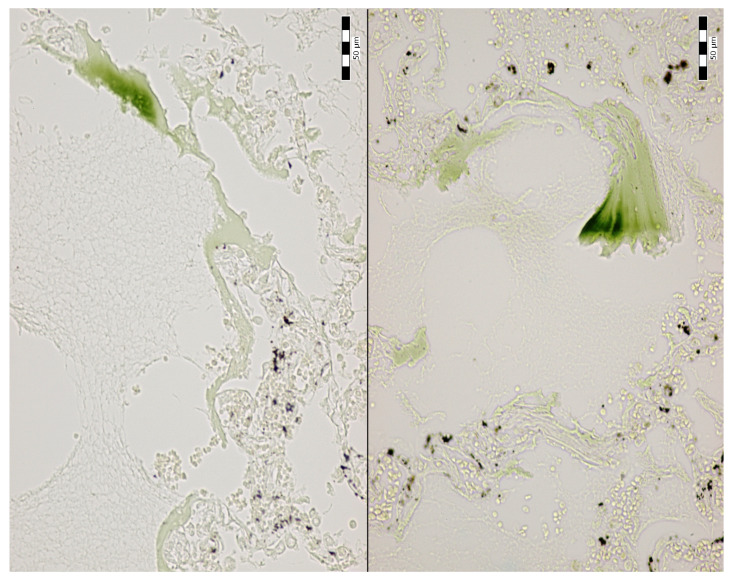
Alveolar bile deposition in unstained sections of the lungs (400x).

**Figure 8:**
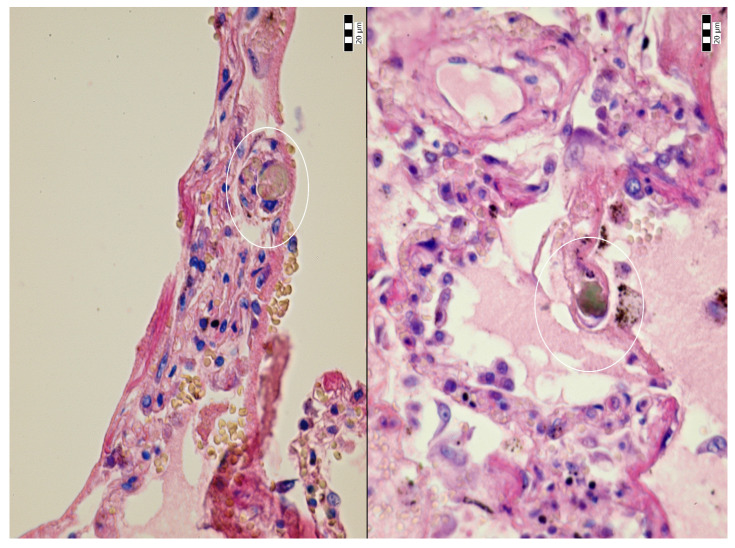
Bile emboli in the pulmonary arterioles (H & E, 600x, circle).

**Figure 9:**
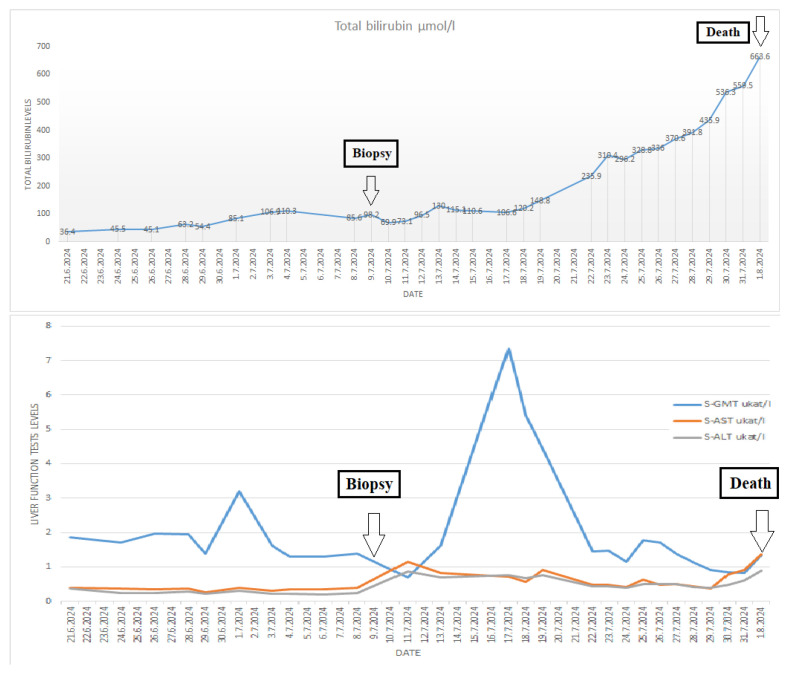
Dynamics of the liver function tests (bilirubin, alanine transaminase/ALT, and aspartate transaminase/AST) during the patient’s hospital stay.

An autopsy was conducted 12 h after the patient’s death. There was well-marked general jaundice of the skin and conjunctivae. The lungs (the right lung weighed 1030 g, the left lung weighed 650 g) had a “shock lung” appearance. The arteries showed only mild atherosclerosis. The enlarged heart weighed 515 g and showed left ventricular hypertrophy. There was no fluid collection in the abdominal cavity and no sign of obstruction or dilatation of the extrahepatic ducts. The liver weighed 1785 g. The left hepatic vein appeared to be surgically ligated on the inferior surface of the liver between the left lobe and the caudate lobe and next to the ligamentum venosum. Histologic examination of the liver revealed intracellular cholestasis highlighted by staining for the presence of bile (Figure 1). No signs of hepatocyte necrosis or tumor infiltration were observed. Microscopic examination of the kidneys revealed cholemic nephrosis, which was highlighted by staining for the presence of bile (Figure 2). Histologic examination of the lungs revealed the acute phase (intra-alveolar edema and hyaline membranes) of diffuse alveolar damage (DAD), which correlated with the clinical presentation of ARDS. Sporadic areas exhibiting the proliferative phase of DAD were observed (Figure 3). Pulmonary infarction was not present. A positive immunohistochemical stain for lambda (Figure 4) and kappa light chains was noted in the pulmonary alveoli, expressing linear positivity in the alveolar walls and above fibrin depositions. The positivity of the lambda light chains was more pronounced than the kappa light chains, which correlates with laboratory test results. The most striking histological finding in the hematoxylin–eosin stained sections of the lung was the deposition of greenish amorphous material in the hyaline membranes, which, in some sections, was seen forming individual “green” membranes (Figure 5). In contrast to the emerald green amorphous material in the sections of the lung stained for the presence of bile, the hyaline membranes showed a yellow coloration (Figure 6). In unstained (deparaffinized) sections, the lung tissue and hyaline membranes were inconspicuous and light green to pale green in color, whereas in hematoxylin–eosin stained sections the greenish amorphous material had a dark green coloration (Figure 7); however, it was not birefringent when exposed to plane-polarized light and did not show autofluorescence. Sporadic bile emboli were spotted in the pulmonary arterioles (Figure 8). The areas with alveolar bile deposition showed no inflammatory reactions. To assess the causative mechanism of alveolar bile deposition, we evaluated the dynamics of the liver function tests (bilirubin, alanine transaminase/ALT and aspartate transaminase/AST) during the patient’s hospital stay (Figure 9). Death was attributed to ARDS complicating the multiple myeloma and idiopathic cholestatic liver disease in a patient with left-sided heart failure.

## 3. Discussion

Risks and unwanted complications are associated with any diagnostic or therapeutic procedure [5]. In this case, a transjugular liver biopsy was performed to elucidate the cause of liver injury associated with elevated liver enzymes, thrombocytopenia, and secondary coagulopathy. The liver biopsy and necropsy revealed intracellular cholestasis. However, no such changes (e.g., necrosis) that would have explained the elevated levels of liver enzymes were observed. Major complications of TJLB occur mostly from capsular perforation and subsequent intraperitoneal hemorrhage [6]. Other reported complications include hepatic hematoma with intraperitoneal hemorrhage, inferior vena cava perforation, renal vein perforation [2], cholangitis [7], hepatic artery thrombosis, a retained guidewire, and massive hemorrhage from the jugular access site [8]. Complications usually occur within three days of the procedure [8]. Intraperitoneal hemorrhage is the most common cause of death following TJLB [2]. Fatal hemobilia resulting from an arteriobiliary fistula is rare [9]. Depending on the bleeding site, the hemorrhage can be managed with arterial [2] or venous embolization [10]. In our case, intraperitoneal bleeding was complicated by hemorrhagic shock appearing on the day of the biopsy; the bleeding was successfully managed with surgical revision of the abdominal cavity.

Bilirubin is the by-product of hemoglobin degradation. Normal serum levels of conjugated bilirubin range from 0.2 to 1 mg/dL (3.4–17.1 µmol/L). Conjugated bilirubin is excreted by hepatocytes into the bile canaliculi and transported to the small intestine in bile. High levels may indicate biliary obstruction, intrahepatic cholestasis, or hepatocellular damage.

Bile pulmonary embolism (BPE) is a rare form of non-thrombotic pulmonary embolism [11]. Bile reaches the lungs through a fistulous communication between the biliary tree and the hepatic venous system. Slow bile flow into a blood vessel may result in a spontaneous closure of the fistula [12]. Bilhemia is bile leakage into the venous system, initially described by Clemens [12] in 1973. In BPE, microscopic examination of the lungs shows the presence of bile in pulmonary arterioles, which can be associated with hepatobiliary diseases or trauma. The first case of BPE following liver aspiration biopsy was described in 1952 [13]. Other cases were documented following percutaneous transhepatic biliary drainage [14,15,16], percutaneous transhepatic cholangiography [17], endoscopic retrograde cholangiopancreatography [18], percutaneous radiofrequency thermal ablation [19], and hepatic microwave ablation [20]. Cases of traumatic BPE following firearm injury [11] and blunt liver trauma [21,22], as well as cases of non-traumatic BPE following acute pancreatitis [23], have also been described in the literature. In our case, alveolar bile deposition was the most striking feature; the pulmonary arterioles were barely affected. An extensive literature search failed to reveal other such cases. Given that the bile deposition dominantly affected the pulmonary alveoli, it is most likely that the amount of bile leaking from the idiopathic unhealed biopsy site into the hepatic veins was sufficient to visibly accumulate in the alveoli without leading to fatal bile pulmonary embolism and acute cor pulmonale. Bilhemia presents with rapidly increasing jaundice and elevated bilirubin levels with no increase in liver enzymes [12], as in the presented case. Elevated bilirubin levels and liver enzymes occurred before TJLB, with a slight decrease occurring the day after the biopsy. Subsequently, the bilirubin levels slightly increased, with a sharp increase noted on the 13th day after the biopsy. Increasing serum bilirubin levels were observed until the patient’s death. During his hospital stay, the patient had not suffered any trauma and did not have any condition which could have resulted in bilhemia (e.g., liver cancer).

Bilhemia without BPE can occur as a complication of medical procedures such as transjugular intrahepatic portosystemic shunt [24] or percutaneous liver biopsy [25]. It is highly probable that impaired healing at the liver biopsy site resulted in a biliovenous fistula on the 13th day after the biopsy. Cholestasis with increased biliary pressure was sufficient to overcome hepatic venous pressure, which led to bile leaking into the venous system and pulmonary alveoli. Biliovenous fistulas can be treated with embolization coils [25]. Given that the patient simultaneously suffered from multiple diseases (left-sided heart failure, multiple myeloma), it is impossible to certainly confirm that the ante mortem diagnosis of biliovenous fistula with subsequent coiling would have prevented the patient’s death. In this case, the biliary embolism was not massive enough to cause immediate bile leakage into the inferior vena cava and pulmonary arteries, which would have led to an immediate bilirubin level increase. In such a case, the patient would have experienced acute dyspnea, which may have resulted in his death. The autopsy would have revealed massive bile emboli in the pulmonary arteries. In our case, bile emboli were spotted only in two pulmonary arterioles.

Normal levels of bile pigments have anti-mutagenic, anti-inflammatory, antioxidant, and immunosuppressive effects, and excess buildup can produce cytopathic effects [26]. Increased serum bilirubin levels are associated with higher mortality rates in patients with ARDS [27], sepsis [28]), and trauma patients [29].

A healthy liver exerts protective effects on the lungs and plays an essential role in lung recovery following lung injury. Patients with liver disease are at significantly higher risk of developing irreversible ARDS [30]. Bile deposition in the lungs may potentiate and accelerate the development of diffuse alveolar damage (DAD) in a patient with multiple myeloma and left-sided heart failure.

This case report is a rare presentation of DAD associated with lambda and kappa light chain depositions in the pulmonary alveoli, resulting in pulmonary insufficiency [31,32]. The differential diagnosis of hyaline membrane disease should include multiple myeloma, which can produce patterns mimicking non-neoplastic hyaline membranes. The presence of hyaline membranes highlighted by hematoxylin and eosin stain is an indicator of both left-sided heart failure and paraprotein accumulation, which can be distinguished only by immunohistochemistry. Postmortem diagnosis of alveolar paraprotein depositions was in keeping with the clinical feature of progressive dyspnea. The presence of paraproteins found in the blood samples of patients with diseases, such as systemic capillary leak syndrome, may induce increased endothelial permeability [33,34] and changes in blood viscosity [35]. In the described case, the circulating paraproteins may have led to pulmonary capillary endothelial damage and facilitated leakage and accumulation of bile in the pulmonary alveoli. To what extent alveolar bile depositions and bilhemia could potentiate lung injury associated with hyaline membrane formation cannot be determined. This case demonstrates that patients with highly compromised health conditions are at greater risk of TJLB complications.

## 4. Conclusions

This case report is a unique presentation of DAD associated with alveolar bile and light chain immunoglobulin depositions in a patient with multiple myeloma and left-sided heart failure, which has never been reported in the literature before. Due to severe thrombocytopenia and secondary coagulopathy, TJLB was performed to differentiate the causes of hepatopathy and elevated liver enzymes. The procedure was complicated by an intraperitoneal hemorrhage. The bleeding was managed expectantly. Significantly elevated serum bilirubin levels occurred on the 13th day after liver biopsy and continued to rise until the patient’s death on the 23rd day after liver biopsy. Impaired healing at the liver biopsy site resulted in alveolar bile deposition. Alveolar bile deposition may be potentiated by hyperviscosity and increased pulmonary capillary permeability due to paraproteinemia. This case report demonstrates that a sudden increase in bilirubin levels after TJLB may indicate the presence of a potentially fatal biliovenous fistula. In cases where no plausible cause of hyaline membranes is noted, the differential diagnosis should include conditions associated with paraproteinemia. The authors report an extremely rare phenomenon, which has never before been described in the scientific literature. The comparison of the pathological and clinical presentation are of paramount importance in diagnosis and treatment of patients with complications following TJLB.

## Data Availability

Primary data are available from the corresponding author upon reasonable request.
